# Dealing with heterogeneity of cognitive dysfunction in acute depression: a clustering approach

**DOI:** 10.1017/S0033291720001567

**Published:** 2021-12

**Authors:** Muriel Vicent-Gil, Maria J. Portella, Maria Serra-Blasco, Guillem Navarra-Ventura, Sara Crivillés, Eva Aguilar, Diego Palao, Narcís Cardoner

**Affiliations:** 1 Mental Health Department, Hospital Universitari Parc Taulí, Institut d'Investigació i Innovació Parc Taulí (I3PT), Universitat Autònoma de Barcelona (UAB), Biomedical Research Networking Center Consortium on Mental Health (CIBERSAM), Parc Taulí 1, 08208 Sabadell, Catalonia, Spain; 2 Department of Psychiatry, Hospital de la Santa Creu i Sant Pau, Biomedical Research Institute Sant Pau (IIB-Sant Pau), Universitat Autònoma de Barcelona (UAB), Biomedical Research Networking Center Consortium on Mental Health (CIBERSAM), Sant Antoni Mª Claret 167, 08025 Barcelona, Catalonia, Spain

**Keywords:** Acute episode, analysis, cluster, cognition, heterogeneity, major depressive disorder

## Abstract

**Background:**

Heterogeneity in cognitive functioning among major depressive disorder (MDD) patients could have been the reason for the small-to-moderate differences reported so far when it is compared to other psychiatric conditions or to healthy controls. Additionally, most of these studies did not take into account clinical and sociodemographic characteristics that could have played a relevant role in cognitive variability. This study aims to identify empirical clusters based on cognitive, clinical and sociodemographic variables in a sample of acute MDD patients.

**Methods:**

In a sample of 174 patients with an acute depressive episode, a two-step clustering analysis was applied considering potentially relevant cognitive, clinical and sociodemographic variables as indicators for grouping.

**Results:**

Treatment resistance was the most important factor for clustering, closely followed by cognitive performance. Three empirical subgroups were obtained: cluster 1 was characterized by a sample of non-resistant patients with preserved cognitive functioning (*n* = 68, 39%); cluster 2 was formed by treatment-resistant patients with selective cognitive deficits (*n* = 66, 38%) and cluster 3 consisted of resistant (*n* = 23, 58%) and non-resistant (*n* = 17, 42%) acute patients with significant deficits in all neurocognitive domains (*n* = 40, 23%).

**Conclusions:**

The findings provide evidence upon the existence of cognitive heterogeneity across patients in an acute depressive episode. Therefore, assessing cognition becomes an evident necessity for all patients diagnosed with MDD, and although treatment resistant is associated with greater cognitive dysfunction, non-resistant patients can also show significant cognitive deficits. By targeting not only mood but also cognition, patients are more likely to achieve full recovery and prevent new relapses.

## Introduction

Research over the last decade has been mainly focused on cognitive performance after a depressive episode (Semkovska et al., 2019) so as to explore difficulties in cognition as an independent facet of clinical manifestation of major depressive disorder (MDD). Some studies report improvements in cognitive performance upon remission of depression (Biringer et al., 2007) whereas others suggest that cognitive impairment persists during clinical remission (Bora, Harrison, Yücel, & Pantelis, 2013; Hasselbalch, Knorr, & Kessing, 2011). The disparity in these results might be explained by the fact that a number of patients do not show any cognitive impairment over the course of the disorder, and others display significant cognitive difficulties even in a non-symptomatic phase. In this regard, a recent study has even found discrete neurocognitive subgroups suggesting the presence of substantial heterogeneity in neurocognitive performance in MDD patients with current affective stability (Pu, Noda, Setoyama, & Nakagome, 2018). It is noteworthy to mention that although patients were in clinical remission, some of them were classified as globally impaired showing moderate to severe cognitive impairment. Therefore, it would be reasonable to think that unresolved cognitive deficits were already present during the acute phase of MDD, from which it could be inferred that different cognitive profiles existed among patients. Despite such a heterogeneity being frequently proposed as an explanation for disparate findings in cognitive performance during a depressive episode (Hammar & Ardal, 2009; Lee, Hermens, Porter, & Redoblado-Hodge, 2012), it has scarcely been investigated. A recent study by our group has already found two distinguishable cognitive profiles in first-episode patients (Vicent-Gil et al., 2018), whereas other reports used mixed samples of mood disorders (Cotrena, Branco, Kochhann, Shansis, & Fonseca, 2016; Iverson, Brooks, Langenecker, & Young, 2011).

Previous studies have described that cognitive dysfunction could be associated with more severe manifestations of the disease (Serra-Blasco & Lam, 2019), such as early/late illness onset, symptom severity (McDermott & Ebmeier, 2009), number of previous depressive episodes (Semkovska et al., 2019) and a higher level of resistance to antidepressant strategies (Murrough et al., 2015; Pimontel et al., 2016; Serra-Blasco et al., 2015). All these studies did not take into account the possible heterogenic cognitive profiles among the included patients, which could have explained the small-to-moderate effect sizes reported so far. Apart from that, other sociodemographic variables, such as years of schooling (Venezia et al., 2018) and age (Dotson, Resnick, & Zonderman, 2008), could also be associated with lower cognitive performance. In fact, two recent studies on clustering analysis of cognitive functioning have shown that the most affected cognitive profile was characterized by poorer general functioning (i.e. poorer intellectual ability; Pu et al., 2018; Vicent-Gil et al., 2018).

Recent studies have reported an association of cognitive dysfunction with worse psychosocial functioning, both at work performance and in social relationships (Clark, DiBenedetti, & Perez, 2016; Cotrena et al., 2016; Evans, Iverson, Yatham, & Lam, 2014). Considering the above, it might be useful to identify those patients with cognitive impairment during an acute episode of depression, to try to cope with cognitive deficits while at the same time improving the patients' psychosocial functioning without having to wait to treat those deficits after clinical remission. So far, only one of the studies mentioned in a sample of first episode of depression has analyzed neuropsychological heterogeneity in the acute stage of the illness revealing two distinguishable cognitive profiles (preserved and impaired clusters) (Vicent-Gil et al., 2018). Some patients were classified as cognitively impaired showing significant deficits in attention/working memory and verbal memory and subtle impairment in executive function, whereas the rest did not show any cognitive deficit. Unfortunately, cognition is not routinely evaluated in all MDD patients [as reported by McAllister-Williams et al. (2017) in the UK], and the presence of cognitive dysfunction in a non-negligible percentage of them still represents an unresolved problem, which derives in increased social and health costs.

Even though there seems to be some evidence about the heterogeneity in cognitive functioning among MDD patients (see Douglas et al., 2018), no studies have attempted to identify subgroups of acute patients considering the factors mentioned above. The aim of the current study is to identify clusters of MDD patients with an acute episode using cognitive, clinical and sociodemographic measures as indicators of grouping. We hypothesize that different cluster groups will emerge based on their cognitive, clinical and sociodemographic characteristics. In addition, we also expect that most cognitively affected patients will show worse psychosocial adaptation.

## Methods

### Participants

A sample of 174 participants aged 18–65 years old was selected from the outpatient unit at the Psychiatry Department of the Hospital Universitari Parc Taulí, from part of a broader project studying the cognitive functioning in major depression (Serra-Blasco et al., 2019). The patients fulfilled the inclusion criteria of a current episode of MDD (DSM-IV-TR criteria). Diagnosis was double checked by two experienced psychiatrists and validated through clinical reports. Exclusion criteria for all participants were the following: (i) presence of any neurological disease, (ii) medical illness with a known impact on cognitive functioning, (iii) intelligence quotient (IQ) <85, (iv) presence of a comorbid axis I diagnosis with the exception of anxiety disorders and dysthymia, (v) past or current substance abuse or (vi) any axis II diagnosis according to the DSM-IV-TR. Participants were on medication at the time of evaluation. Patients were invited to participate in this cross-sectional study, which included a clinical and neuropsychological assessment conducted by experienced research neuropsychologists. The study was set following the principles of the Declaration of Helsinki and was approved by the Research Ethics Board of the Institut d'Investigació i Innovació Parc Taulí (I3PT) at Hospital Universitari Parc Taulí. All participants gave their written informed consent after a full and comprehensive explanation of the study.

### Clinical and demographic assessment

Clinical and demographic variables were obtained during a semi-structured interview, which covered age, sex, years of schooling, age at illness onset, number of episodes and duration of illness. Medication use at the time of evaluation was categorized as: no medication, monotherapy with antidepressants, antidepressants plus benzodiazepines and combination of antidepressants with one or more psychotropic drugs (e.g. antipsychotics, lithium and anticonvulsants). Depressive symptomatology was assessed using the 17-item Hamilton Depression Rating Scale (HDRS-17; Bobes *et al*. 2003; Hamilton, 1960). The Maudsley Staging Method (MSM; Fekadu, Wooderson, Markopoulou, & Cleare, 2009) was administered to assess the level of treatment resistance. MSM includes information about duration and severity of depression, antidepressant treatments, augmentation strategies and electroconvulsive therapy providing two categories: non-resistant (scores 3–6) and resistant (scores 7–15).

### Neuropsychological assessment

The neuropsychological battery included the following tests: Digit Span of the Wechsler Adult Intelligence Scale version IV (WAIS-IV; Wechsler, 2008); Rey Auditory Verbal Learning Test (RAVLT; Rey, 1941); Digit Symbol Substitution Test (DSST; WAIS-IV); Trail Making Test Part A (TMT-A), and Trail Making Test Part B (TMT-B; Tombaugh, 2004); Wisconsin Card Sorting Test (WCST; Heaton, 1981); similarities subtest (WAIS-IV); semantic verbal fluency test (category fluency; Benton & Hamsher, 1976; Peña-Casanova et al., 2009) and phonemic verbal fluency test (PMR, adapted for Spanish speaking population; Casals-Coll *et al*., 2013; Peña-Casanova *et al*., 2009). Premorbid intelligence (estimated IQ) was assessed with Vocabulary Subtest of the WAIS-IV and it was used to compare cognitive profiles after the clustering analysis.

### Functional assessment

Functioning Assessment Short Test (FAST; Rosa et al., 2007) was used to evaluate autonomy, occupational functioning, cognitive functioning, financial issues, interpersonal relationships and leisure time. The scores range from 0 to 72 with higher values indicating more disability. A score of ⩾12 represents a mild to severe functional impairment (Bonnín et al., 2018).

### Data analyses

Data were analyzed using the Statistical Package for Social Sciences (SPSS), version 21. Neuropsychological raw scores were converted to *z*-scores using normative data. To reduce the number of neuropsychological variables (Miskowiak et al., 2017; Mur, Portella, Martínez-Arán, Pifarré, & Vieta, 2007), cognitive domains were defined using a principal component analysis (PCA). The number of retained components was decided upon the resulting scree plot, admitting eigenvalues above or close to 1 (based on theoretically-driven decision). These components (cognitive domains) were then rotated and used for a cluster analysis.

To identify homogeneous subgroups of patients, a two-step clustering analysis was carried out based on cognitive domains, stage of treatment-resistance (MSM), depressive symptomatology (HDRS-17), number of depressive episodes, age and years of schooling. This two-step analysis is designed to use categorical and continuous variables in large samples. It represents an extra value to previous studies as other relevant variables such as treatment response can be included. During the first step, subjects are preclustered into small subgroups using a sequential-clustering approach. In the second step, subclusters from the first are entered as inputs and grouped into the best number of clusters according to a hierarchical-clustering method (Norusis, 2011). The determination of the optimal numbers of clusters is based on the Akaike Information Criterion (AIC) and the log-likelihood distance, taking as the best solution the large ratio of AIC changes and the large ratio of distance measures. The final model is based on different criteria (Nylund, Asparouhov, & Muthén, 2007): (i) the highest cohesion and separation of the resulted clusters measured with the Silhouette's index, (ii) the best clinical coherence and (iii) an adequate number of patients in each cluster to facilitate statistical analyses of comparison. Also, as a final result, the analyses provide a ranking of the importance of each predictor entered in the model. The greater the importance measure, the more relevant the variable is considered in the formation of the cluster.

Demographic, clinical and functional variables were analyzed among resulting clusters in each group of patients by means of one-way analysis of variances or chi-square when appropriate, and effect-sizes were reported, as well.

## Results

The PCA of neuropsychological data extracted four orthogonal dimensions that corresponded to four cognitive domains and explained a 74% of cumulative variance. The four cognitive domains were: (i) Attention/Working Memory domain, which included the forward and backward Digit Span; (ii) Verbal Memory domain, composed of RAVLT first trial, immediate recall and delayed recall; (iii) Executive Function domain, with TMT Part A, TMT Part B, DSST and number of categories from WCST and (iv) Verbal Ability domain which included PMR, semantic fluency and similarities. See online Supplementary Table 1 with a brief summary of outcome measures for each test.

**Fig. 1.**
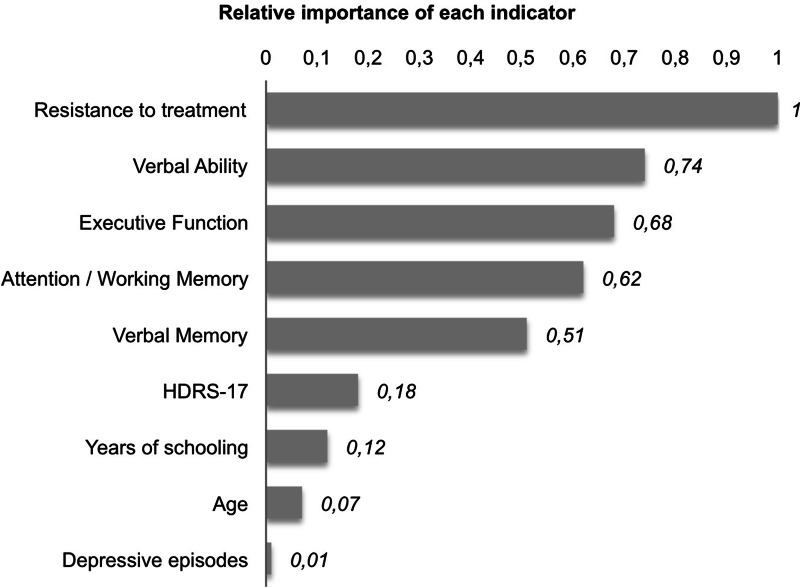
Clustering summary: relative importance of each indicator.

The two-step clustering analysis resulted in a three-cluster solution, which was selected as being the most optimal one based on a fair Silhouette's index (equal to 0.3, see online Supplementary Fig. 1), on the clinical interpretability from previous studies, and on the number of patients in each cluster. As can be observed from Fig. 1, the stage of treatment resistance obtained the highest relevance for clustering with a predictor importance of 1.0, followed by verbal ability, executive function, attention/working memory and verbal memory with values ranging between 0.8 and 0.5. Depressive symptomatology, years of schooling, age and number of depressive episodes obtained the lowest relevance for clustering. Table 1 presents the centroids for the predictors, mean values of quantitative variables and percentage distribution for the categorical variable treatment resistance.

**Table 1.** Clustering summary: centroids

	C1	C2	C3	Combined
Categorical indicators				
MSM non-resistant^a^	80%	0%	20%	100%
MSM-resistant^a^	0%	74.2%	25.8%	100%
Quantitative indicators (means)				
Verbal ability	−0.46	−0.71	−1.86	−0.88
Executive function	−0.45	−0.79	−1.83	−0.9
Attention/working memory	−0.51	−0.74	−1.71	−0.88
Verbal memory	−0.49	−1.07	−1.92	−1.04
HDRS-17	17.15	19.33	23.25	19.38
Years of schooling	10.5	9.97	8.05	9.74
Age	52.16	54.97	51.95	53.18
Depressive episodes	2.19	2.41	2.43	1.35

MSM, Maudsley Staging Method; HDRS, Hamilton Depression Rating Scale.

a MSM non-resistant (scores 3–6), MSM-resistant (scores 7–15).

**Fig. 2.**
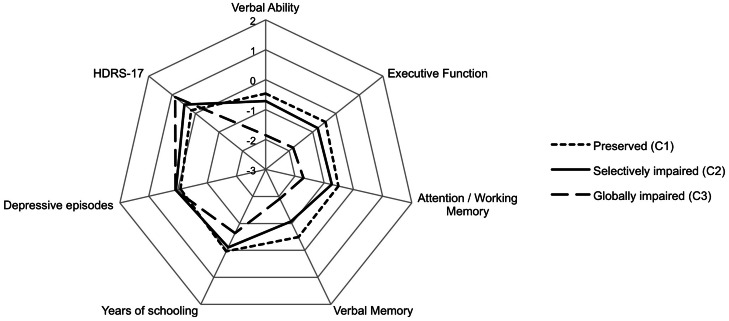
Radar chart for the distribution of the indicators of the model. HDRS-17: Hamilton Depression Rating Scale.

Figure 2 illustrates a radar chart that shows the profile of the clusters within the different predictors of the model (quantitative variables were transformed into *z*-scores for a better comprehension of the figure). The first cluster (C1) was classified as cognitively preserved (*n* = 68, 39%) and it was characterized by a sample of non-resistant patients. The second cluster (C2) included 66 patients (38%) classified as selectively impaired and all patients were treatment-resistant. And cluster 3 (C3) included globally impaired patients (*n* = 40, 23%) with significant deficits in all neurocognitive domains, being 23 patients classified as resistant (58%) and 17 as non-resistant (42%).

Tables 2 and 3 display comparisons among the three clusters for demographic, clinical and functional variables and for cognitive tests. The three clusters differed in terms of cognitive performance (see online Supplementary Fig. 2), in which C1 patients (cognitively preserved) scored within normal range in almost all the tests (with the exception of number of categories). C2 patients (selectively impaired) obtained scores below 1s.d. in specific tests evaluating memory and executive functioning. And C3 patients (globally impaired) showed significant alterations in almost all the neuropsychological tests (with *z*-scores ranging between −1 and −2). *Post-hoc* comparisons of C3 (globally impaired patients) to C1 and C2 showed large effect sizes in all cognitive variables (Cohen's *d* > 0.8, with the exception of first trial in RAVLT in the contrast C2 *v.* C3). With regard to functional assessment, significant differences were also observed, in which globally impaired patients showed the worst outcomes.

**Table 2.** Mean scores (standard deviation) for demographic and clinical variables across clusters

	Clusters			Statistics		*Post-hoc*			Cohen's *d*^a^		
	C1 Preserved (*n* = 68)	C2 Selectively impaired (*n* = 66)	C3 Globally impaired (*n* = 40)	*F* or χ^2^	*p*	C1 *v.* C2	C1 *v.* C3	C2 *v.* C3	C1 *v.* C2	C1 *v.* C3	C2 *v.* C3
Age (years)	52.16 (8.62)	54.97 (6.22)	51.95 (7.97)	2.94	0.056	0.104	1.0	0.150	0.37	0.03	0.42
Sex, *female n* (%)	49 (72.1)	40 (60.6)	33 (82.5)	5.9	0.052	0.16	0.22	0.018	0.12	0.12	0.23
Educational level (years)	10.5 (3.36)	9.97 (3.29)	8.05 (2.03)	8.29	<0.001^b^	0.96	<0.001^b^	0.006	0.16	0.88	0.7
Estimated IQ, *T-score*	51.68 (8.27)	49.52 (7.49)	43.3 (3.67)	17.63	<0.001^b^	0.246	<0.001^b^	<0.001^b^	0.27	1.31	1.05
Age at illness onset (years)	42.13 (12.12)	42.17 (9.76)	38.8 (12.23)	1.35	0.262	1.0	0.423	0.418	0.00	0.27	0.30
Number of episodes, *n*	2.19 (1.42)	2.41 (1.2)	2.43 (1.47)	0.57	0.566	1.0	1.0	1.0	0.17	0.17	0.01
Illness duration (months)	127.3 (122.07)	171.68 (110.38)	171.63 (145.52)	2.67	0.073	0.118	0.221	1.0	0.38	0.33	0.00
HDRS-17, *total score*	17.15 (6.03)	19.33 (6.56)	23.25 (4.9)	12.99	<0.001^b^	0.110	<0.001^b^	0.004	0.35	1.11	0.68
Comorbidities, *n* (%)	20 (29.4)	21 (31.8)	18 (45.0)	0.294	0.230	0.762	0.101	0.173	0.03	0.16	0.13
Anxiety disorders, *n* (%)	3 (4.4)	6 (9.1)	6 (15.0)	0.361	0.164	0.279	0.055	0.352	0.09	0.19	0.09
Dysthymia, *n* (%)	17 (25.0)	17 (25.8)	12 (30.0)	0.349	0.840	0.920	0.571	0.635	0.01	0.05	0.05
MSM, resistant *n* (%)	0 (0)	66 (100)	23 (58)	134.9	<0.001^b^	<0.001^b^	<0.001^b^	<0.001^b^	1.0	0.68	0.56
FAST, *total score*	36.49 (13.39)	42.68 (14.19)	47.93 (13.48)	9.17	<0.001^b^	0.03	<0.001^b^	0.176	0.45	0.85	0.38
Current medication, *n* (%)				18.98	<0.001^b^	<0.001^b^	0.01	0.482	0.38	0.29	0.07
AD	30 (44.1)	7 (10.6)	8 (20)								
AD + BZD	22 (32.4)	22 (33.3)	11 (27.5)								
AD + Others	14 (20.6)	37 (56.1)	20 (50)								
Medication-free	2 (2.9)	0 (0)	1 (2.5)								

IQ, intelligence quotient; HDRS, Hamilton Depression Rating Scale; MSM, Maudsley Staging Method; FAST, Functional Assessment Short Test; AD, antidepressant; BZD, benzodiazepines; others, antipsychotics; lithium or anticonvulsants.

a Cramer's *V* for categorical variables.

b Analysis of variance or χ^2^ tests statistically significant after applying Bonferroni correction for multiple comparisons (*p* < 0.004).

**Table 3.** Mean scores (standard deviation) for cognitive variables across clusters

	Clusters			Statistics		*Post-hoc*			Cohen's *d*		
	C1 Preserved (*n* = 68)	C2 Selectively impaired (*n* = 66)	C3 Globally impaired (*n* = 40)	*F*	*p*	C1 *v.* C2	C1 *v.* C3	C2 *v.* C3	C1 *v.* C2	C1 *v.* C3	C2 *v.* C3
**Attention/working memory**	−0.51 (−0.88)	−0.74 (0.62)	−1.71 (0.45)	54.41	<0.001^a^	0.071	<0.001^a^	<0.001^a^	0.30	1.72	1.79
Forward digit (WAIS-IV)	−0.7 (0.66)	−0.89 (0.77)	−1.9 (0.59)	41.32	<0.001^a^	0.331	<0.001^a^	<0.001^a^	0.26	1.92	1.47
Backward digit (WAIS-IV)	−0.33 (0.79)	−0.6 (0.75)	−1.5 (0.54)	34.80	<0.001^a^	0.091	<0.001^a^	<0.001^a^	0.35	1.73	1.38
**Verbal memory**	−0.49 (0.74)	−1.07 (0.72)	−1.92 (0.85)	44.52	<0.001^a^	<0.001^a^	<0.001^a^	<0.001^a^	2.14	1.79	1.08
First trial (RAVLT)	−0.65 (0.81)	−0.92 (0.78)	−1.49 (1.14)	11.22	<0.001^a^	0.251	<0.001^a^	0.005	0.34	0.85	0.58
Immediate recall (RAVLT)	−0.56 (0.87)	−1.26 (1.0)	−2.38 (0.98)	46.42	<0.001^a^	<0.001^a^	<0.001^a^	<0.001^a^	0.75	1.96	1.13
Delayed recall (RAVLT)	−0.27 (0.88)	−1.02 (0.92)	−1.89 (0.96)	40.53	<0.001^a^	<0.001^a^	<0.001^a^	<0.001^a^	0.83	1.76	0.93
**Executive function**	−0.45 (0.72)	−0.79 (0.63)	−1.83 (0.48)	60.9	<0.001^a^	0.007	<0.001^a^	<0.001^a^	0.50	2.26	1.86
TMT part A	−0.27 (0.97)	−0.5 (0.87)	−1.76 (0.82)	37.61	<0.001^a^	0.426	<0.001^a^	<0.001^a^	0.25	1.66	1.49
TMT part B	−0.01 (1.05)	−0.61 (0.93)	−1.98 (0.62)	38.66	<0.001^a^	0.003	<0.001^a^	<0.001^a^	0.60	2.28	1.73
DSST (WAIS-IV)	−0.29 (0.88)	−0.66 (0.74)	−1.82 (0.76)	47.01	<0.001^a^	0.022	<0.001^a^	<0.001^a^	0.46	1.86	1.55
Categories (WCST)	−1.11 (0.82)	−1.34 (0.76)	−1.84 (0.44)	12.01	<0.001^a^	0.240	<0.001^a^	0.003	0.29	1.11	0.81
**Verbal ability**	−0.46 (0.64)	−0.71 (0.67)	−1.86 (0.48)	68.06	<0.001^a^	0.06	<0.001^a^	<0.001^a^	0.38	2.47	1.97
Phonemic fluency (PMR)	−0.45 (0.77)	−0.67 (0.73)	−1.68 (0.66)	37.58	<0.001^a^	0.255	<0.001^a^	<0.001^a^	0.29	1.72	1.45
Semantic fluency (Animals)	−0.6 (0.86)	−0.98 (0.83)	−1.97 (0.78)	34.31	<0.001^a^	0.025	<0.001^a^	<0.001^a^	0.45	1.67	1.23
Similarities (WAIS-IV)	−0.34 (1.0)	−0.49 (1.02)	−1.93 (0.82)	37.12	<0.001^a^	1.0	<0.001^a^	<0.001^a^	0.15	1.74	1.56

WAIS: Wechsler Adult Intelligence Scale, RAVLT: Rey Auditory Verbal Learning Test, TMT: Trail Making Test, DSST: Digit Symbol Substitution Test, WCST: Wisconsin Card Sorting Test.

a Analysis of variance or χ^2^ tests statistically significant after applying Bonferroni correction for multiple comparisons (*p* < 0.003).

## Discussion

This work explores the existence of empirical clusters for MDD taking into account patients' cognitive performance, stage of treatment resistance, depressive symptomatology, number of depressive episodes, age and years of schooling. Treatment resistance was the variable with the highest importance of clustering, closely followed by cognitive performance (verbal ability, executive function, attention/working memory and verbal memory). The rest of variables obtained the lowest importance on identifying distinct subgroups of patients. Three empirical clusters were determined: cluster 1, characterized by non-resistant patients with preserved cognitive performance; cluster 2, composed of resistant patients with selective impairment and cluster 3, grouped by resistant and non-resistant patients with a global cognitive impairment. As hypothesized, the latest was related to worse clinical and psychosocial outcomes. These findings may indicate the existence of different subgroups of patients, determined by clinical variables – as well-established in the literature – and by cognitive symptoms, which have not received enough attention for decades and may be underlying poor outcomes.

To our knowledge, this study is the first to show different cognitive profiles during an acute phase of the illness, considering not only cognitive performance but also clinical and sociodemographic factors, which are likely to have contributed to divergent results in the last few decades. Hammar and Ardal (2009) already suggested that no single cognitive functioning profile could characterize all depressed patients, and that not all patients were to be impaired in the same degree during the acute phase (Hammar & Ardal, 2009). The existence of such heterogeneity among mood disorders' cognitive functioning is supported by previous cluster analyses, especially in bipolar disorder (Burdick et al., 2014; Cotrena, Branco, Ponsoni, Shansis, & Fonseca, 2017; Lima et al., 2019; Solé et al., 2016). These studies found three-cluster solutions based on cognitive performance, corresponding to intact or preserved, selectively impaired and globally impaired patients. Only two studies have been carried out clustering analysis with exclusively MDD patients, and their findings point toward cognitive heterogeneity along the different stages of the disorder. One included patients in partial remission and reported three clusters (Pu et al., 2018) in accordance with the above-mentioned studies, and the other was centered into first-episode patients and showed two clusters, one preserved and one impaired (Vicent-Gil et al., 2018). These previous studies, except for the one with first-episode patients, endorse our current findings of a subgroup of intact or mostly preserved patients, of a globally impaired subgroup with a general cognitive affectation, and of a subgroup with specific domains impaired. Although some of the studies (Burdick et al., 2014; Pu et al., 2018) claim to have found ‘discrete neurocognitive subgroups’, this may be straightforward for preserved and globally impaired patients, because these individuals can be easily detected even in clinical settings. By contrast, the selectively impaired subjects may not constitute a clearly discrete neurocognitive subgroup given that other characteristics may interact with cognition making difficult to detect specific cognitive alterations. At this point, our current study highlights the importance of adding clinical information to the clustering, as it may be crucial for a more comprehensive classification of patients, by capturing other factors that may blur those patients in C2.

Treatment resistance was the most important variable in the clustering process, which embraces a lack of response (and/or remission) and greater severity of clinical symptoms. The totality of participants in the cognitively preserved cluster was non-resistant and similarly, the 100% of the participants in the selectively impaired cluster were resistant. In the group of globally impaired patients, however, up to 42% of patients were non-resistant. A possible explanation is that alterations of memory and executive function are more specifically related to treatment resistance (Pimontel et al., 2016; Rao et al., 2019), as observed in C2, in consistence with previous evidence of hippocampus and prefrontal cortex alterations in treatment-resistant depression (Ge et al., 2019). Thus, a global alteration of cognitive function may be reflecting a different phenotype, in which the main characteristic would be cognitive impairment and not exclusively linked to treatment resistance – this may not be unreasonable as the majority of antidepressant treatments do not target cognitive symptoms. These results might indicate that although treatment resistance would be associated with greater cognitive impairment, the existence of various cognitive profiles has to be taken into account beyond the usual clinical variables, as non-resistant patients can still display cognitive symptoms. In fact, even though all patients of the study were acutely depressed, 39% showed no cognitive impairment. These findings also help to explain the small-to-moderate effects – depending on the domain – of cognitive dysfunction in previous studies that compare patients with MDD with healthy controls, considering the apriorism of cognitive homogeneity among MDD patients may have led to such disperse results. The current study, which is data-driven, demonstrates the relevant role of cognitive functioning in major depression, pointing at the existence of potential cognitive dysfunction in a high percentage of patients who will require more tailored treatments.

Socio-demographic variables have a low relative importance in the cluster formation (Fig. 1). However, years of schooling and IQ significantly differ among clusters, where the most impaired patients – cluster 3 – had on average 2 years less of schooling and almost one standard deviation less of IQ than the cognitively preserved group. Differences in IQ could be tautological as positive associations have been described between IQ and cognitive performance. However, in clinical practice it is usually observed that patients with normal or intact premorbid IQ show relevant cognitive impairment, and the other way around (patients with limited IQ who do not show any cognitive deficits). Although globally impaired patients had the lowest IQ in this study, their intellectual ability fell within normality, and their cognitive performance was below normality (1.5s.d. below, on average). All the above-mentioned variables, which are normally used as proxies for cognitive reserve, showed a significant importance in previous studies of cluster analysis (Pu et al., 2018; Vicent-Gil et al., 2018). And cognitive reserve itself has specifically shown to moderate the relationship between mood and cognition (Opdebeeck et al., 2017). Thus, cognitive reserve should not be neglected when assessing MDD patients.

By considering both cognitive and other illness-related variables, the three clusters may reflect variations of the disorder due to differences in underlying pathophysiology, in response to treatment or in illness trajectories, and not merely in cognitive subdivisions on a linear continuum (Carruthers, Van Rheenen, Gurvich, Sumner, & Rossell, 2019). Consequently, the clinical implication of the present results refers to the necessity of considering cognitive functioning in clinical settings in all patients diagnosed with major depression. First, clinicians should seriously contemplate addressing cognitive symptoms when a given patient begins to show resistance to treatment, as cognitive difficulties may be related to worse treatment outcomes. Therefore, the intervention should be directed not only to clinical symptoms, but also to cognitive dysfunction. Second, 42% of patients who respond adequately to antidepressants may also present a global cognitive dysfunction. In clinical practice, some patients with a good response to antidepressant treatment complain about a lack of complete recovery and of difficulties to perform daily activities, partly due to their perceived cognitive problems. Detecting such unidentified cognitive difficulties is of great importance as they are associated with worse psychosocial functioning (Cambridge, Knight, Mills, & Baune, 2018; Knight & Baune, 2018) and with the low rates of recovery (Groves, Douglas, & Porter, 2018). Hence, treating cognitive dysfunction together with clinical symptomatology in an active episode of depression could probably result in a better response to treatment. Different pharmacological strategies (e.g. vortioxetine, duloxetine, modafinil or erythropoietin), non-pharmacological approaches (cognitive remediation or aerobic exercise) and neurostimulation interventions have been shown to be effective in the treatment of cognitive dysfunction while improving psychosocial functioning and quality of life (Salagre et al., 2017; Zuckerman et al., 2018).

The current study was subject to some limitations. First, due to cross-sectional design it was not possible to assess the long-term stability of the cognitive profiles. Second, the sample included outpatients treated in a specialized clinical setting, and may not comprise worldwide clinical practice as mild outpatients may be underrepresented. Third, the mean age of the sample was older than other studies, which could have facilitated the inclusion of patients with cognitive deficits caused by other conditions (such as neurodegenerative processes). In any case, the presence of a neurological condition was an exclusion criterion which was strictly evaluated. Fourth, although two-step clustering analysis is one of the most robust techniques to classify individuals (as it combines *k*-means and hierarchical approaches), the generalizability of the findings is one of the main drawbacks as the results are very sample-specific. The lack of external replication with an independent dataset represents a limitation of these results. In any case, our findings are fairly similar to the scarce literature. Fifth, individual scores were corrected with demographic-adjusted norms from a similar population, which might affect the interpretation of the present findings; therefore, future research should consider the cognitive heterogeneity within the norm samples. To overcome this limitation, composite scores for cognitive performance were used as a more objective patient's cognitive performance outcome compared to the use of single tests (Miskowiak et al., 2017). Sixth, the variety of impaired cognitive domains within the ‘selectively impaired cluster’, when compared to other studies, reflects another kind of heterogeneity that cannot be resolved with cluster analysis, as the solution depends on the samples used in each study. However, by using a two-step clustering, we detected two clear and extreme subgroups (i.e. preserved and globally impaired) together with a subgroup of selective impairment, in which other factors, beyond cognition, help to better characterize them. Finally, concomitant medication could be a possible confounder because of its side effects on cognition. But due to clinical reasons and ethical concerns, it was not adequate to discontinue the medication.

In conclusion, the current study shows the existence of distinguishable subgroups in a sample of acute depressed patients, in which treatment resistance and cognitive performance are relevant factors to take into account. The current design provides evidence for the heterogeneity of cognitive dysfunction in MDD. Therefore, future clinical research should consider the existence of potential cognitive dysfunction in all MDD patients, to tailor new strategies to achieve a full clinical and functional recovery and to prevent new relapses.

## Supplementary material

For supplementary material accompanying this paper visit https://doi.org/10.1017/S0033291720001567. click here to view supplementary material
