# Supplementary material for: Dealing with heterogeneity of cognitive dysfunction in acute depression: a clustering approach
Source: Psychol Med. 2020 Jun 1;51(16):2886–94. doi: 10.1017/S0033291720001567 (PMC8640365; doi:10.1017/S0033291720001567)
Supplement: Supplementary file 1 [file S0033291720001567sup.zip › S0033291720001567sup002.docx]

| **Supplementary Table 1**. *Neuropsychological Assessment* | | | | |
| --- | --- | --- | --- | --- |
| Cognitive Domains* | Neuropsychological Test | Subtest | Outcome measure | Cognitive Function |
| **ATTENTION**  **WORKING MEMORY** | Digit Test (Wechsler Adult Intelligence Scale, version-IV) | Digit forward | Sum of correct responses | Selective attention |
|  |  | Digit backward | Sum of correct responses | Verbal Working Memory |
| **VERBAL MEMORY** | Rey Auditory Verbal Learning Test (RAVLT) | First Trial | Sum of trial 1 | Short term auditory-verbal memory |
|  |  | Immediate recall | Sum of trial 1 to 5 | Verbal learning |
|  |  | Delayed recall | Sum of trial 6, after 25-30’ | Long-term auditory-verbal memory |
| **EXECUTIVE FUNCTION** | Trail Making Test (TMT) | Part A (TMT-A) | Time taken to complete the test | Cognitive processing speed |
|  |  | Part B (TMT-B) | Time taken to complete the test | Set-shifting ability |
|  | Digit Symbol Substitution Test (DSST; WAIS-IV) | - | Sum of correct responses | Motor speed, attention and visuoperception |
|  | Wisconsin Card Sorting Test (WCST) | - | Number of categories completed | Categorization and shifting abilities |
| **VERBAL ABILITY** | Category Fluency | Animals | Sum of correct responses | Semantic verbal fluency |
|  | Phonemic Fluency | PMR | Sum of correct responses | Phonemic verbal fluency |
|  | Similarities subtest (WAIS-IV) | - | Sum of correct responses | Logical thinking and verbal abstract reasoning |
| *Cognitive domains were based on a Principal Component Analysis (PCA). | | | | |
