# Supplementary material for: Dealing with heterogeneity of cognitive dysfunction in acute depression: a clustering approach
Source: Psychol Med. 2020 Jun 1;51(16):2886–94. doi: 10.1017/S0033291720001567 (PMC8640365; doi:10.1017/S0033291720001567)
Supplement: Supplementary file 1 [file S0033291720001567sup.zip › S0033291720001567sup003.docx]

| **Supplementary Table 2**. *Auto-Clustering Statistics* | | | | |
| --- | --- | --- | --- | --- |
| Number of  clusters | AIC | AIC  change^a^ | Ratio of AIC  changes^b^ | Ratio of distance  measures^c^ |
| 1 | 1381.944 |  |  |  |
| 2 | 1194.600 | -187.343 | 1.000 | 1.684 |
| 3 | 1097.197 | -97.413 | .520 | 1.900 |
| 4 | 1062.024 | -35.163 | .188 | 1.326 |
| 5 | 1043.853 | -18.171 | .097 | 1.183 |
| 6 | 1033.767 | -10.087 | .054 | 1.424 |
| 7 | 1036.805 | 3.039 | -.016 | 1.168 |
| 8 | 1044.286 | 7.481 | -.040 | 1.174 |
| 9 | 1055.689 | 11.402 | -.061 | 1.096 |
| 10 | 1069.066 | 13.377 | -.071 | 1.151 |
| 11 | 1085.153 | 16.087 | -.086 | 1.057 |
| 12 | 1102.208 | 17.056 | -.091 | 1.010 |
| 13 | 1119.439 | 17.230 | -.092 | 1.104 |
| 14 | 1138.248 | 18.809 | -.100 | 1.005 |
| 15 | 1157.136 | 18.888 | -.101 | 1.082 |
| ^a^The changes are from the previous number of clusters in the table.  ^b^The ratios of changes are relative to the change for the two cluster solution.  ^c^The ratios of distance measures are based on the current number of clusters against the previous numbers of clusters. | | | | |
