# Supplementary figures and images for: Dealing with heterogeneity of cognitive dysfunction in acute depression: a clustering approach
Source: Psychol Med. 2020 Jun 1;51(16):2886–94. doi: 10.1017/S0033291720001567 (PMC8640365; doi:10.1017/S0033291720001567)

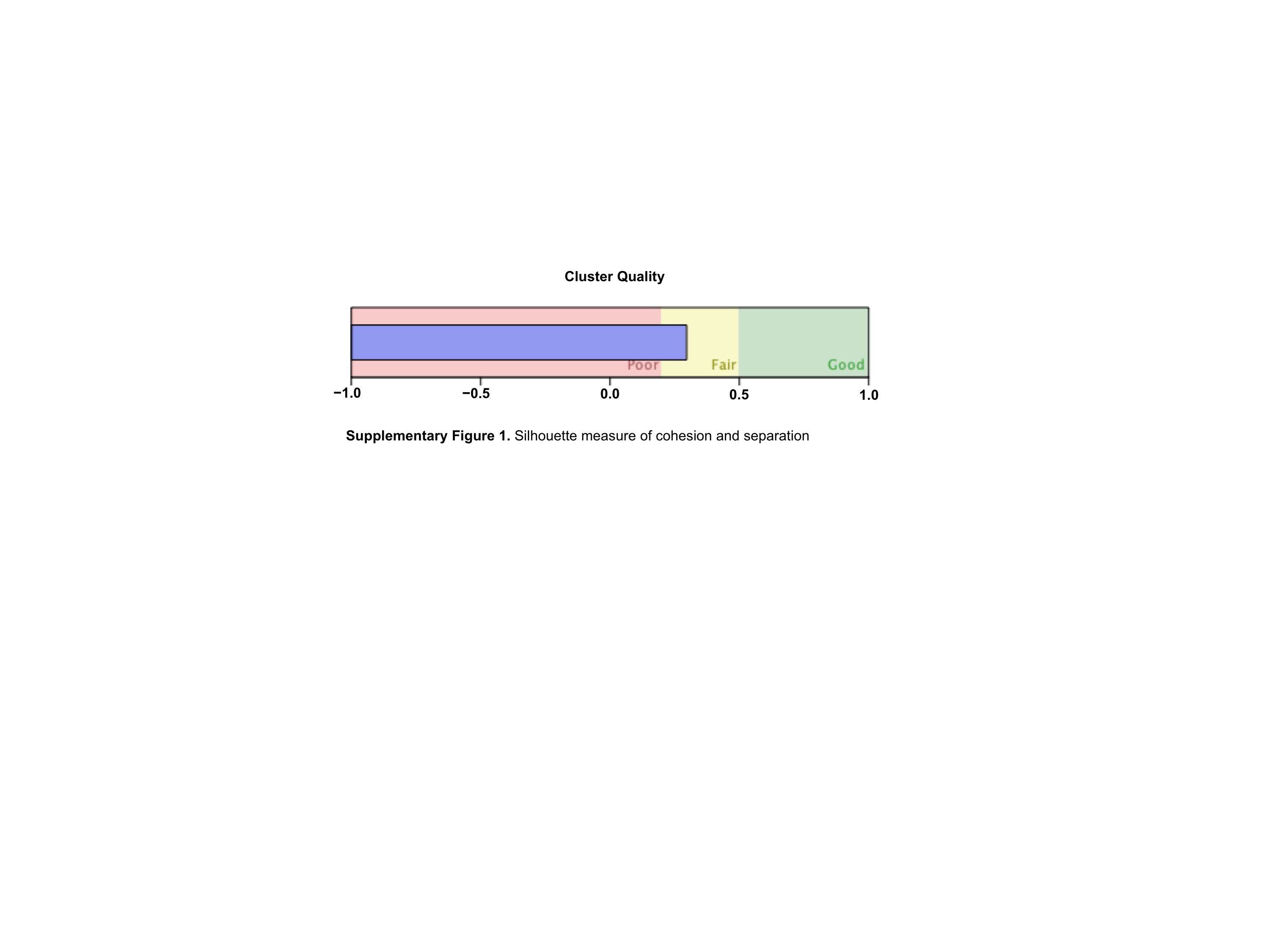

Supplement: Supplementary file 1 [file S0033291720001567sup.zip › S0033291720001567sup004.tiff]
